# The clinicopathological and immunohistochemical features of breast carcinomas with signet-ring-cell differentiation

**DOI:** 10.1186/s12957-023-03074-x

**Published:** 2023-06-19

**Authors:** Jie Zheng, Junlin Liu, Wan Yang, Jia Yao, Jiao Guo, Changqing Liu

**Affiliations:** 1 Department of Pathology, The First People’s Hospital of Jingmen, Hubei, China; 2 Department of Gastroenterology, The First People’s Hospital of Jingmen, Hubei, China

**Keywords:** Carcinoma, Signet ring cell, Breast neoplasms, Immunohistochemistry, Pathology

## Abstract

**Background:**

This study investigated the clinicopathological features, immunophenotypic characteristics and differential diagnosis of primary breast carcinomas with signet ring cell differentiation, as well as differences in the traits of signet ring-like cell mucin.

**Methods:**

A total of five cases of primary breast cancer diagnosed with signet ring cell differentiation and treated at The First People’s Hospital of Jingmen from January 2016 to December 2021 were collected. HE, immunohistochemical staining, and AB-PAS staining were used for the analysis.

**Results:**

Although we strictly selected all the primary breast cancer cases with signet ring cell differentiation, there were differences in the arrangement of the cells and the grading of nuclei. Our immunohistochemical results showed that the ER was consistently strongly positive, and the PR expression was not consistent, while all the cases of HER2 were negative. CK7 was negative in one case, and CK20 and CK5/6 were not expressed in all the cases. The mucin MUC1 was positive and showed two patterns. MUC2 was strongly positive in all the cases. All the cases were negative for CDX2, SATB2, PAX8, TTF-1, and Napsin A, while the positive expression of COX2, Villin, and WT-1 was not constant. One case expressed neuroendocrine markers. The expression level of Ki67 was between 10 and 30%. AB (pH 2.5)-PAS staining revealed that the intracellular mucus contained more cells with neutral mucus, while the extracellular mucus was mainly acidic.

**Conclusion:**

We found that histological morphology, cell morphology, and nuclear grading differentiate among different cases. The immunohistochemical characteristics of primary breast cancers diagnosed with signet ring cell differentiation are helpful for identification. The differences in the expression patterns of mucins may be related to unfavorable clinicopathological factors, but their usefulness as a prognostic marker remains to be further understood. The heterogeneity of cell mucus, the differentiation of tumor cells, and the phenotypic changes of tumors also need further study.

## Background

Primary signet ring cell carcinoma of the breast (SRCC) was first described by Saphir as a mucinous carcinoma [1], and to date, few cases of primary signet ring cell carcinoma of the breast have been reported. The diagnostic criteria for SRCC have not been strictly defined either, since marked signet ring cell differentiation can occur in invasive lobular carcinoma, nonspecialized invasive carcinoma, and other specialized types; therefore, the WHO classification of breast tumors does not classify SRCC as a distinct disease entity, and the WHO changed the name of SRCC to carcinoma with signet ring cell differentiation. For convenience, we still use SRCC in the following description.

However, tumors with signet ring-like cells can occur in many organs of the body, such as the digestive tract, especially the common gastric signet ring cell carcinoma, the salivary glands, lungs, ovaries, etc. [2–4]. Therefore, we need more specific markers for each tumor site to distinguish whether cancer with signet ring cell differentiation has metastasized to the breast. Aberrant expression of the membrane-bound form of mucin, MUC1, and the secreted form of mucin, MUC2, may be associated with cancer growth, differentiation, transformation, and invasion [5]. However, the role of mucin in primary breast cancer with signet ring-like cell differentiation remains to be further investigated.

In this article, we analyzed breast cancer cases with signet ring cell differentiation. The histological morphology of each case was carefully described. We evaluated the staining pattern of several common immunohistochemical markers, including markers commonly used in breast cancer including Estrogen receptor (ER), progesterone receptor (PR), human epidermal growth factor receptor 2 (HER-2), gross cystic disease fluid protein15 (GCDFP-15), and GATA binding protein 3.

(GATA-3); epithelial and myoepithelial expression patterns including CK7, CK20, and CK5/6; and especially the relative specific markers of the digestive system, including CDX2, cyclooxygenase 2(COX-2), Specially rich in AT sequence binding proteins (SATB-2), and Villin. Lung adenocarcinoma-related markers were evaluated including Thyroid transcription factor-1 (TTF-1) and Napsin A, and ovarian cancer-related markers including pair box gene 8 (Pax-8) and Wilm’s tumor gene 1(WT-1). The expression of neuroendocrine factors was also described. Differences in morphological and biological behaviors among different cases of breast cancer with signet ring cell differentiation and differences in immunohistochemical expression between breast cancer with signet ring cell differentiation and signet ring cell carcinomas derived from other sites have been discovered and identified. At the same time, the expression patterns of mucins, including MUC1 and MUC2, were found to be different from those of the surrounding concomitant cancer tissues. By Alcian Blue (pH 2.5)–Periodic Acid–Schiff (AB-PAS) staining, it was found that the characteristics of the mucus in the signet ring cells in the same case were also different.

## Methods

### Case selection

We collected a total of 5 cases of primary invasive breast carcinoma diagnosed with signet ring cell differentiation from the Department of Pathology of The First People’s Hospital of Jingmen from January 2016 to December 2021.These cases were strictly selected. According to the definition of WHO 2012, cancer with signet ring cell differentiation must be characterized by rich intracellular mucus, which squeezes the nucleus to one side to produce the characteristic signet ring cell morphology [6]. Based on this definition, we re-reviewed all mucinous adenocarcinomas and invasive lobular carcinomas and confirmed these five cases. We collected complete clinicopathological data, including tumor size, lymph node metastasis, vascular invasion, recurrence, and death. We reviewed hematoxylin and eosin-stained histological specimens from each case to confirm the presence of SRC within the tumor. The population of SRCs in each case was assessed quantitatively by two pathologists (RO and TS) who independently determined the percentage of SRCs among the total number of tumor cells evaluated in at least 20 high-power fields focusing on the SRC-rich areas. Nuclear atypia was graded by combining four nuclear features, including (1) enlargement, (2) distinct nucleolus, (3) hyperchromasia, and (4) pleomorphism, and was expressed as grade 1: none or 1 of the features, grade 2: 2 or 3 of the features, and grade 3: all of the features [7].

The clinical data extracted from medical charts and pathology reports were reviewed. This study was conducted in accordance with the principles embodied in the Declaration of Helsinki (revised in Brazil in 2013). Patient consent was obtained for the use of the clinical samples for research purposes according to the regulations defined by the Ethics Committee of The First People’s Hospital of Jingmen (Batch number: 202202013).

### Hematoxylin and eosin (HE) staining

Place the slices in sequence into xylene I for 10 min, xylene II for 10 min, anhydrous ethanol I for 5 min, anhydrous ethanol II for 5 min, 95% alcohol for 5 min, 90% alcohol for 5 min, 80% alcohol for 5 min, 70% alcohol for 5 min, and distilled water for washing. Hematoxylin stained cell nucleus Slice and stain with Harris hematoxylin for 3–8 min, wash with tap water, differentiate with 1% hydrochloric acid alcohol for a few seconds, rinse with tap water, return to blue with 0.6% ammonia water, and rinse with running water. Slice and stain in eosin staining solution for 1–3 min. Place the slices in order of 95% alcohol I for 5 min-95% alcohol II for 5 min-anhydrous ethanol I for 5 min-anhydrous ethanol II for 5 min-xylene I for 5 min-xylene II for 5 min, and dehydrate them until transparent. Remove the slices from xylene and air dry them slightly, and seal them with neutral gum. The catalog number and company of all materials are summarized in Table 1.

**Table 1:** The catalog number and company of all materials

Reagents	Source	Catalog number
xylene	Zhongtian Wuhan, CHN	20220406X
Anhydrous ethanol	Zhongtian Wuhan, CHN	20220404E
95% alcohol	LIRCON Shandong, CHN	20220201B
Hematoxylin	Baso Zhuhai, CHN	C220206
Eosin	Baso Zhuhai, CHN	C220203
Ethylenediaminetetraacetic acid (EDTA) antigen retrieval solution	MXB Fuzhou, CHN	220407s425j
Hydrogen peroxide (H2O2) solution	LIRCON Shandong, CHN	20221208B
Phosphate-buffered saline (PBS)	MXB Fuzhou, CHN	22,120,602
Alucian blue (pH2.5)-Periodic acid solution	Baso Zhuhai, CHN	C210301

### Immunohistochemistry

Three-micron-thick mammary paraffin sections were selected, placed in a 70 °C oven for 30 min, and then deparaffinized and rehydrated in a series of xylenes and graded alcohols. The treated sections were placed in a boiled ethylenediaminetetraacetic acid (EDTA) antigen retrieval solution (pH 9.0) for 3 min and then immersed in a 3% hydrogen peroxide (H2O2) solution to block endogenous peroxide enzymatic activity. Sections were washed with phosphate-buffered saline (PBS) and incubated overnight at 4 °C with primary antibodies against ER, PR, Her-2, GCDFP-15, GATA-3, CK7, CK20, CK5/6, MUC-1, MUC-2, Villin, COX2, SATB2, WT1, Pax8, TTF1, NapsinA, Cyn, CgA, CD56, and Ki67. The characteristics and dilutions of the primary antibodies are summarized in Table 2.

**Table 2:** Characteristics of primary antibodies

Antibody	Source	Host	Catalog number	Clone	Dilution
Estrogen receptor (ER)	DAKO Carpinteria, CA	Mouse monoclonal	41,432,445	1D5	1:1
Progesterone receptor (PR)	DAKO Carpinteria, CA	Mouse monoclonal	2209280502i	PGR636	2:1
Human epidermal growth factor receptor 2 (HER-2)	MXB Fuzhou, CHN	Rabbit monoclonal	2203080701b	MXR001	1:1
Gross cystic disease fluid protein15 (GCDFP-15)	MXB Fuzhou, CHN	Mouse monoclonal	220,208,071	23A3	Ready-to-use
GATA binding protein 3 (GATA-3)	MXB Fuzhou, CHN	Mouse monoclonal	210,916,136	L50-823	1:3
Cytokeratin 7 (CK7)	DAKO Carpinteria, CA	Mouse monoclonal	220,116,049	OV-TL12/30	1:3
Cytokeratin20 (CK20)	DAKO Carpinteria, CA	Mouse monoclonal	2203150057b	Ks20.8	Ready-to-use
Cytokeratin5/6 (CK5/6)	DAKO Carpinteria, CA	Mouse monoclonal	220,301,048	D5/16 B4	1:1
Cyclin Dependent Kinase 4 (CDK4)	MXB Fuzhou, CHN	Rabbit monoclonal	220407077f	EP180	1:2
Mucin1 (MUC-1)	DAKO Carpinteria, CA	Mouse monoclonal	210,325,191	MRQ-17	Ready-to-use
Mucin2 (MUC-2)	DAKO Carpinteria, CA	Mouse monoclonal	210,325,192	MRQ-18	Ready-to-use
Villin	DAKO Carpinteria, CA	Mouse monoclonal	220,504,117	1D2C3	1:1
CDX-2	DAKO Carpinteria, CA	Mouse monoclonal	2201110631q	DAK-CDX2	Ready-to-use
Specially rich in AT sequence binding proteins (SATB-2)	MXB Fuzhou, CHN	Rabbit monoclonal	2111030750e	EP281	Ready-to-use
Cyclooxygenase 2 (COX-2)	DAKO Carpinteria, CA	Rabbit monoclonal	2201260549f	SP21	1:1
Wilm's tumour gene (WT-1)	DAKO Carpinteria, CA	Mouse monoclonal	B21040803	6F-H2(1,2)	1:1
pair box gene (Pax-8)	DAKO Carpinteria, CA	Mouse monoclonal	220,112,155	MRQ-50	1:2
Thyroid transcription factor-1 (TTF-1)	DAKO Carpinteria, CA	Mouse monoclonal	2203150599b	8G7G3/1	Ready-to-use
NapsinA	DAKO Carpinteria, CA	Mouse monoclonal	220,310,150	MRQ-60	1:3
Synapsin (Syn)	DAKO Carpinteria, CA	Mouse monoclonal	220,302,111	DAK-Synap	2:1
Chromogranin A (CgA)	MXB Fuzhou, CHN	Rabbit monoclonal	220,411,046	SP12	1:1
Neural cell adhesion molecules (CD56)	MXB Fuzhou, CHN	Mouse monoclonal	220,112,034	MX039	1:1
Ki67	DAKO Carpinteria, CA	Mouse monoclonal	220,419,222	SP6	2:1
Epidermal growth factor receptor (EGFR)	DAKO Carpinteria, CA	Mouse monoclonal	220,228,131	UMAB95	Ready-to-use

### Alcian Blue (pH 2.5)–Periodic Acid–Schiff (PAS) staining

The sections were routinely dewaxed in water and washed with distilled water. Alcian blue staining solution (pH 2.5) was then added dropwise for 20 min. Distilled water was used to remove the excess staining solution, and excess water was wiped off the slides. The periodic acid solution was added dropwise to oxidize for 10 min, rinsed with distilled water, and the excess water was shaken off. Then, Schiff’s reagent was added dropwise for 15 min, rinsed with running water, and the excess water was shaken off. Then, the hematoxylin was stained for 3 min, rinsed with running water, returned to blue, dehydrated and cleared in the usual steps, and sealed with neutral gum.

## Results

### Clinicopathological profiles of breast carcinoma with signet ring cell differentiation

The five patients with primary breast cancer with signet ring cell differentiation were all female, aged 53–86 years, with a median age of 56 years. Table 3 shows the representative clinicopathological features. There were 2 cases of tumors located in the outer quadrant of the left breast, 2 cases in the outer quadrant of the right breast, and 1 case in the inner quadrant of the right breast. The breasts of case 1 were accompanied by pain, the breasts of case 5 were accompanied by redness, swelling, pain, and the nipples secreted pus, and the other cases had no pain, itching, redness, swelling, or nipple discharge. The tumor size ranged from 2.6 to 9.5 cm, with an average diameter of 5.12 cm. The tumor boundary was not clear, the cut surface was gray and solid, and the texture was hard. Cases 1 and 5 had lymph node metastasis and vascular invasion. During follow-up thus far, case 1 died, case 5 relapsed, and the rest had no relapse or death.

**Table 3:** Clinicpathological variables of patients with breast cancer containing signet ring cells

Case	Age	Tumor site	Histological type	Tumor size (cm)	Nuclear grade	SRC population (%)	LN status	LVI	Recurrence	Follow-up (months)	Status
1	86	R-UOQ	IDC	9.5	1	80	+	+	+	8	Dead
2	53	R-UOQ	ILC	5	3	20	−	−	−	68	Alive
3	56	L-UOQ	IDC	2.6	1	80	−	−	−	52	Alive
4	60	R-IQT	MC	4	2	50	−	−	−	39	Alive
5	54	L-UOQ	ILC	4.5	1	10	+	+	+	14	Alive

*Abbreviations*: *SRC* signet ring cell, *LN* lymph node, *LVI* lymphovascular invasion, *R* right, *L* left, *UOQ* upper outer quadrant, *IQT* inner quadrant transition

### Microscopic features of conventional HE staining in breast cancer with signet ring cell differentiation

**Fig. 1:**
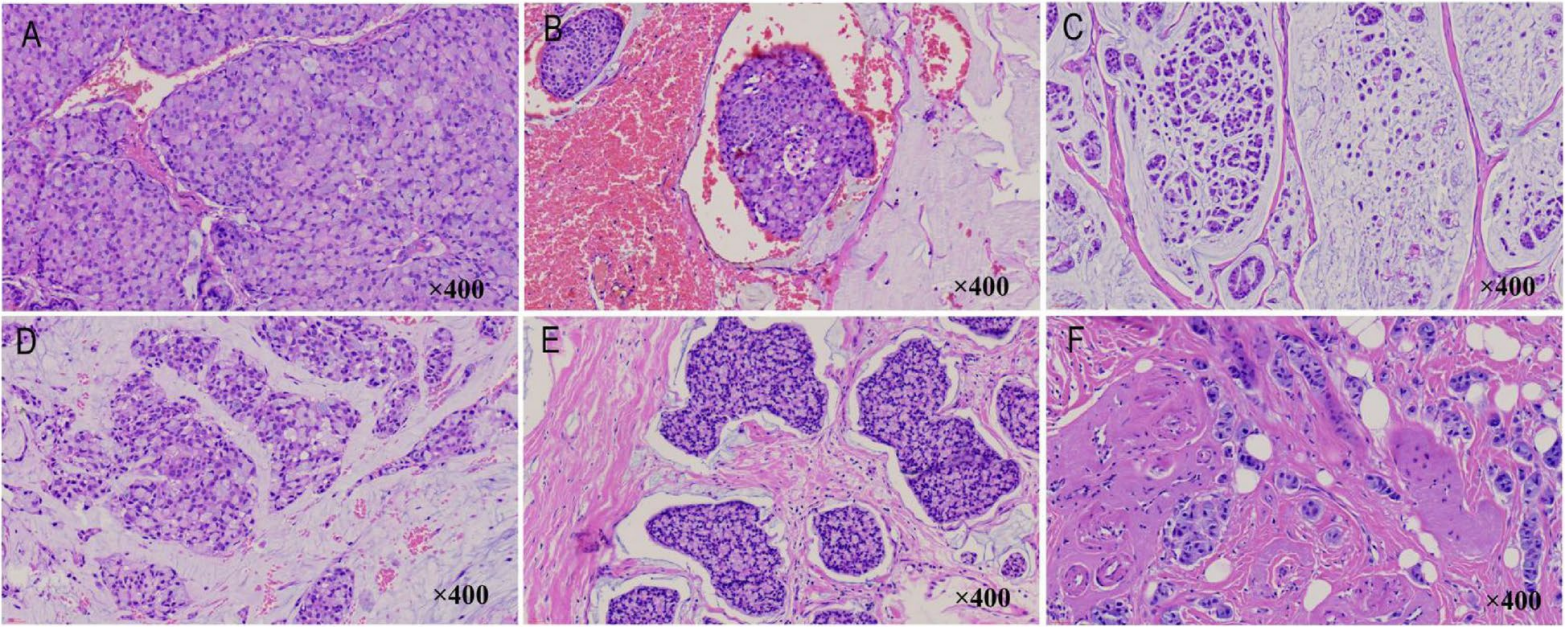
Histologic features in breast cancer with signet ring cell differentiation (hematoxylin and eosin, magnification × 400). **A** In case 1, signet ring-like cancer cells have abundant intracellular mucus, the nucleus is squeezed and displaced. Some nuclei are round, oval, or even crescent-shaped, resembling fat vacuoles. **B** Cancer cell clusters can be seen in the blood vessels of the local area in case 1. **C** In case 2, the signet-ring-like cancer cells had less intracellular mucus, slightly deviated nuclei, greater nuclear atypia, deeper staining, some cells were crescent-shaped. **D** In case 3, the intracellular mucus was abundant, the nucleus was squeezed and displaced, and some nuclei were round, oval, and crescent-shaped. **E** In case 4, signet ring-like cancer cells have abundant intracellular mucin, eccentric nuclei, round, irregular, or crescent-shaped nuclei. **F** In case 5, signet ring-like cancer cells have abundant intracellular mucus, eccentric nuclei, round, fusiform or comma-shaped nuclei

Representative histopathological findings are shown in Fig. 1. In case 1, signet ring cell carcinoma accounted for approximately 80%, and the rest were invasive ductal carcinomas. Signet ring-like cancer cells had abundant intracellular mucus, and the nucleus was not deeply stained. The nucleus was squeezed and displaced. Some nuclei were round, oval, or even crescent-shaped, resembling fat vacuoles (Fig. 1A). The nuclei were graded as grade 1 (Table 3). A large number of signet ring cells aggregated into clusters and the mucus between the cancer cells clustered into a mucus lake. Cancer cell clusters could be seen in the blood vessels of the local area (Fig. 1B). In case 2, signet ring cell carcinoma accounted for approximately 20%, and the rest were invasive lobular carcinomas. The signet-ring-like cancer cells had less intracellular mucus than case 1, slightly deviated nuclei, greater nuclear atypia, and deeper staining, some cells were crescent-shaped, and the nuclei were graded as grade 3 (Table 3). The cells were loosely arranged in strands or as single cells floating in the mucus. There were slender fibers in the middle divided into separate pool-like structures (Fig. 1C). In case 3, signet ring cell carcinoma accounted for approximately 80%, and the rest were invasive ductal carcinomas. In the same signet-ring-like cancer cells, the intracellular mucus was abundant, the nucleus was squeezed and displaced, and some nuclei were round, oval, and crescent-shaped. The cancer cells were arranged in cords and clumps surrounded by a myxoid matrix. Wide fibrous septa were observed (Fig. 1D). In case 4, signet ring cell carcinoma accounted for approximately 50%, and the rest were mucinous adenocarcinoma. Signet ring-like cancer cells had abundant intracellular mucin, eccentric nuclei, round, irregular, or crescent-shaped nuclei, and grade 2 nuclei (Table 3). The cells were tightly arranged and distributed in the mucus or fibrous stroma (Fig. 1E). In case 5, signet ring cell carcinoma accounted for approximately 10%, and the rest were invasive lobular carcinomas. Signet ring-like cancer cells had abundant intracellular mucus, eccentric nuclei, round, fusiform, or comma-shaped nuclei, and grade 1 nuclei (Table 3). The cancer cells were distributed in small groups in the fibrous stroma and adipocytes (Fig. 1F).

### Immunohistochemical findings in breast cancer with signet ring cell differentiation

**Fig. 2:**
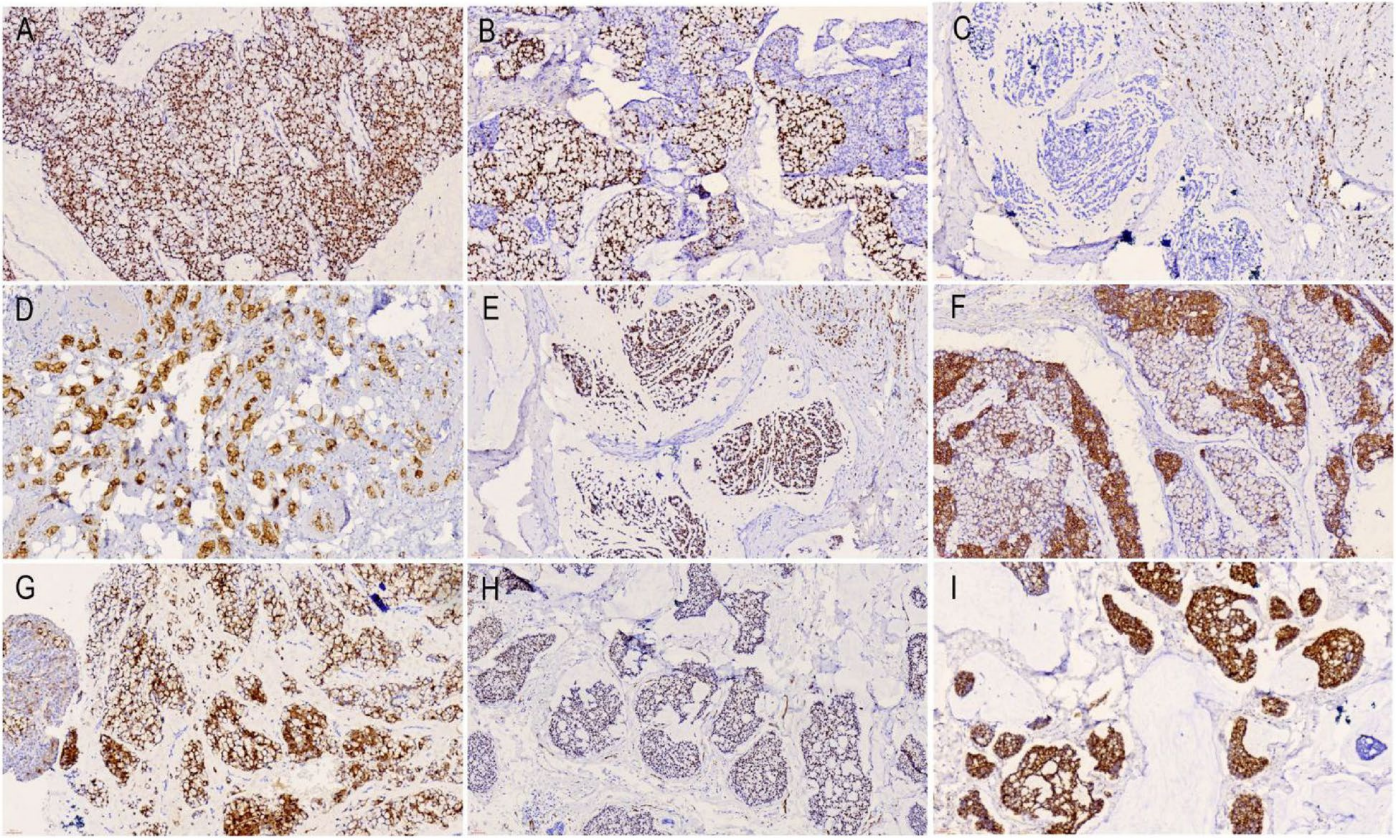
Immunohistochemical Findings in breast cancer with signet ring cell differentiation (magnification × 200). **A** ER is constantly expressed and more than 90% of cancer cells are strongly positive. **B** PR expression in case 1 reaches 90%. **C** PR expression of case 2 is negative. **D** GCDFP15 was expressed in case 5. **E** GATA3 was strongly positive in all cases. **F** CK7 was positive and was down-regulated in signet-ring-like cells compared to surrounding invasive ductal carcinoma cells. **G** Villin expression was positive in case 3. **H** WT-1 was positive in cases 4. **I** CgA were positive in case 4

Immunohistochemical subcellular localization patterns and expression levels are shown in Table 4 and Fig. 2, respectively. ER, PR, and HER2 are often used for molecular typing of breast cancer. In all the cases of breast cancer with signet ring cell differentiation, ER was constantly expressed, and more than 90% of the cancer cells were strongly positive (Fig. 2A). PR expression was not constant, the positive expression of case 1 (Fig. 2B) and case 4 reached 90%, the expression of case 2 and case 5 (Fig. 2C) was negative, and only approximately 10% of the cancer cells in case 3 were positive, while HER2 was negative in all the cases (Table 4). Therefore, according to breast cancer molecular typing, case 1 and case 4 were classified as luminal type A, while case 2, case 3, and case 5 were classified as luminal type B. GCDFP15 was only expressed in case 1 and case 5 (Fig. 2D), and the rest were negative. GATA3 was strongly positive in all the cases with good specificity (Fig. 2E).

**Table 4:** Immunohistochemical results of the present five patients with breast cancer with signet ring cell differentiation

Protein	Patient 1	Patient 2	Patient 3	Patient 4	Patient 5
ER	+ + + (90%)	+ + + (90%)	+ + + (90%)	+ + + (90%)	+ + + (90%)
PR	+ + + (90%)	-	+ + + (10%)	+ + + (90%)	-
HER2	-	-	-	-	-
GCDFP15	+	-	-	-	+
GATA3	+	+	+	+	+
CK7	+	+	-	+	+
CK20	-	-	-	-	-
CK5/6	-	-	-	-	-
MUC1	+	+	+	+	+
MUC2	+	+	+	+	+
Villin	-	-	+	-	-
CDX2	-	-	-	-	-
COX2	-	-	-	+	-
SATB2	-	-	-	-	-
WT-1	+	-	-	+	-
PAX8	-	-	-	-	-
TTF-1	-	-	-	-	-
NapsinA	-	-	-	-	-
Syn	-	-	-	+	-
CgA	-	-	-	+	-
CD56	-	-	-	+	-
Ki67	+ (10%)	+ (25%)	+ (30%)	+ (15%)	+ (20%)

*IHC* immunohistochemistry, *ER* estrogen receptor, *PR* progesterone receptor, *HER2* human epidermal growth factor receptor 2, *GCDFP-15* gross cystic disease fluid protein-15, *SATB2* special AT-rich sequence-binding protein 2, *ER and PR* +  +  + *(%)* proportion of strongly positive cells, *Ki67* + *(%)* proportion of positive cells

Among epithelial and myoepithelial markers, CK7 was positive in case 1, case 2, case 4, and case 5 and was downregulated in the signet-ring-like cells in case 1 compared to the surrounding invasive ductal carcinoma cells (Fig. 2F). The remaining markers CK20 and CK5/6 were not expressed in all the cases (Table 4).

**Fig. 3:**
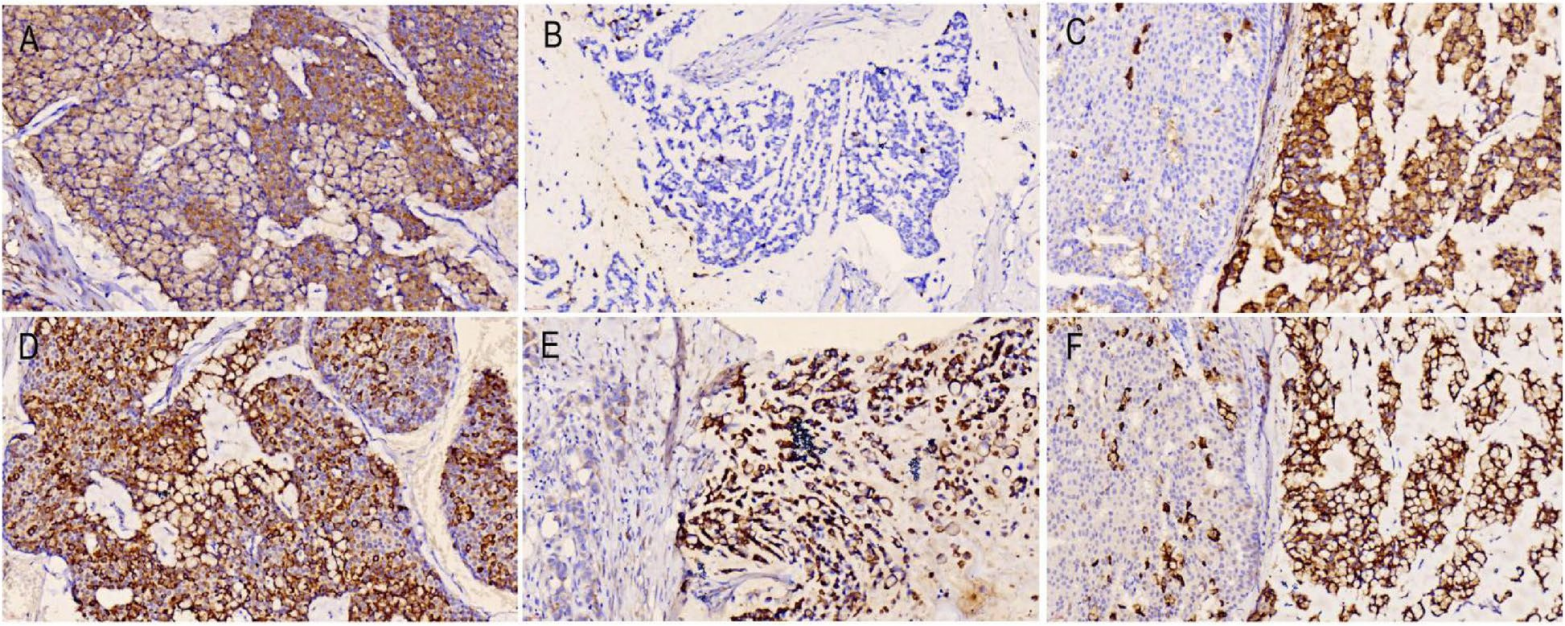
Immunohistochemical expression of MUC1 and MUC2 (magnification × 400). **A** the expression of MUC1 was localized in the mucous cavity of signet ring-like cells, and the expression intensity was weak. **B** MUC1 was only positive in individual tumor cells. **C** MUC1 was expressed in the cell lumen, and the expression around the cell membrane was strongly positive. **D**–**F** MUC2 was strongly expressed, and was significantly enhanced compared with peripheral carcinoma

MUC1 and MUC2, two mucin markers, were positively expressed in all the cases, but the expression patterns were different among the cases. In case 1, the expression of MUC1 was localized in the mucous cavity of the signet ring-like cells, and the expression intensity was weak (Fig. 3A). In case 2, MUC1 was only positive in the individual tumor cells, which was significantly downregulated compared with the surrounding invasive lobular carcinoma (Fig. 3B). In case 3, MUC1 was expressed in the cell lumen, and the expression around the cell membrane was strongly positive, which was significantly higher than that in the surrounding ductal carcinoma (Fig. 3C). In case 4, MUC1 was expressed in the cytoplasm, which was enhanced compared with mucinous carcinoma. In case 5, MUC1 was expressed in the cell lumen, the expression around the cell membrane was strongly positive, and the expression was significantly enhanced compared with the surrounding lobular carcinoma. MUC2 was strongly expressed in all the cases and was significantly enhanced compared with peripheral invasive ductal carcinoma, lobular carcinoma, or mucinous carcinoma (Fig. 3D–F).

Villin, CDX2, COX2, and SATB2 are specific markers derived from gastrointestinal adenocarcinoma, including signet ring cell carcinoma, and are often used to identify tumors from other systems, including the breast, lung, and the female reproductive system. In breast cancers with signet ring cell differentiation, CDX2 and SATB2 expression was negative in all five cases (Table 4), while the Villin expression was positive in case 3 (Fig. 2G) and the COX2 expression in case 4.

The two markers, WT-1 and PAX8, are commonly used to differentiate ovarian cancer from other tumors of epithelial origin. PAX8 was negative in all five cases, while WT-1 was positive in cases 1 and 4 (Fig. 2H) and negative in the other cases. Two markers, TTF-1 and Napsin A, are undoubtedly specific and sensitive indicators for lung adenocarcinoma, and they were negative in five cases of breast cancer with signet ring cell differentiation (Table 4).

Syn, CgA, and CD56 were expressed to varying degrees in the tumors with neuroendocrine differentiation. All three markers were positive in case 4 (Fig. 2I), while all other cases were negative. Ki67, a cell proliferation index marker, has been widely used in the evaluation of breast cancer prognosis and lymph node metastasis. In the five cases of breast cancer with signet ring cell differentiation, the expression level was between 10 and 30% (Table 4).

### Histochemical staining findings

**Fig. 4:**
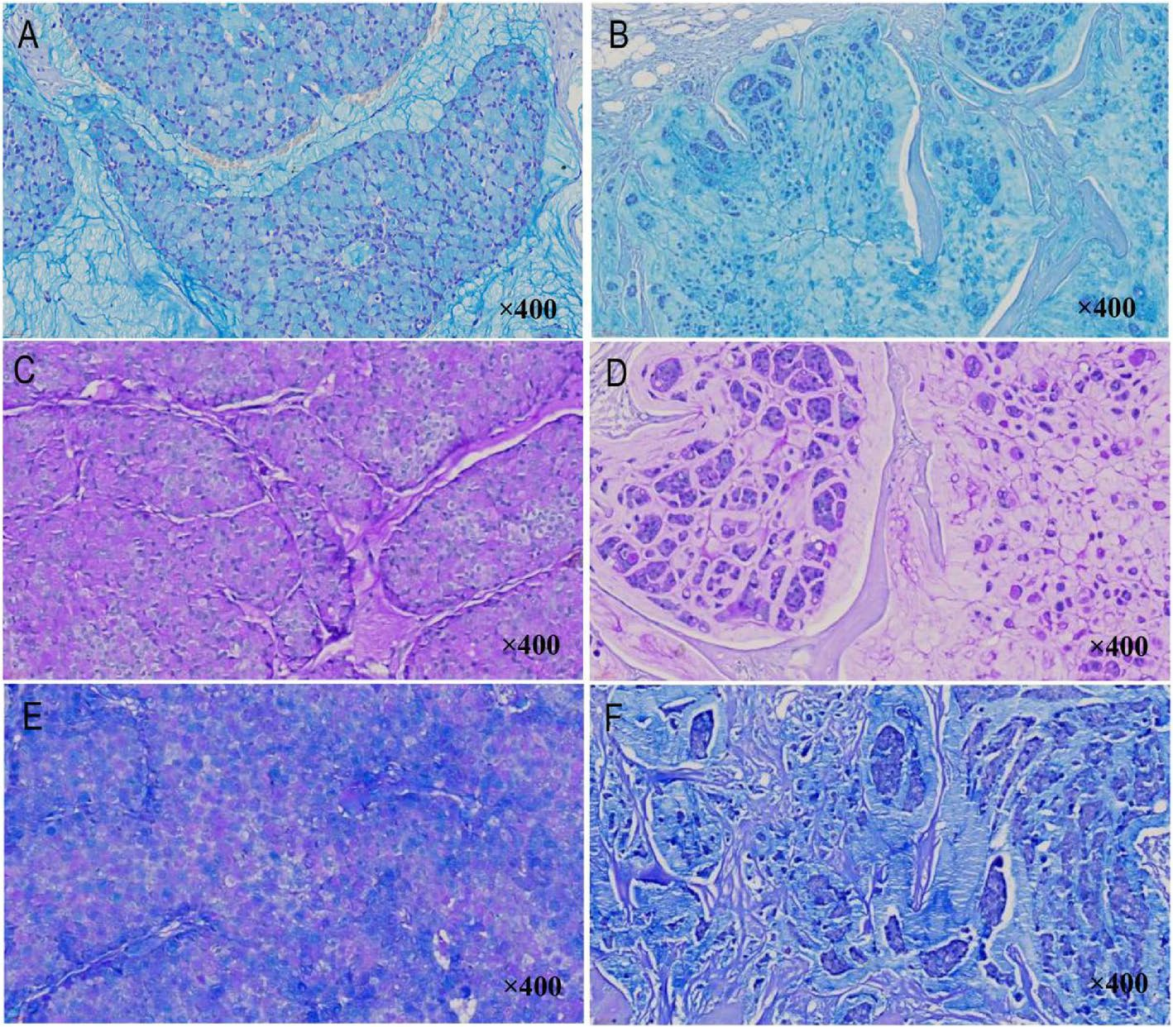
Histochemical staining findings (magnification×400). **A**,**B** Acidic mucus was stained with AB (pH 2.5) and turned blue. **C**,**D** Nutral mucus was stained with PAS, and turned red. **E**,**F** Mixed type mucus was purplish red after staining with AB (pH 2.5)-PAS

AB (pH 2.5) staining, PAS staining, and AB (pH 2.5)-PAS staining were performed on all the cases (as shown in Fig. 4). Acidic mucus was stained with AB (pH 2.5) and turned blue (Fig. 4A, B), and neutral mucus was stained with PAS (Fig. 4C, D) and turned red. Mixed-type mucus was purplish red after staining with AB (pH 2.5)-PAS (Fig. 4E, F). AB (pH 2.5) staining and PAS staining showed that all the cases contained acidic mucus and neutral mucus, both intracellularly and extracellularly, while the AB (pH 2.5)-PAS staining found that some of the cells in all of the cases contained mixed mucus. Overall, the intracellular mucus contained more cells with neutral mucus, while the extracellular mucus was dominated by acidic mucus.

## Discussion

In clinical practice, signet ring cell carcinoma mostly occurs in the gastrointestinal tract. Primary breast signet ring cell carcinoma (SRCC) is very rare. In 2003, the World Health Organization (WHO) classified breast cancer and classified SRCC as the latter “other types” of mucinous carcinoma (special type cancer) and other tumors with rich mucus. In 2012, the WHO changed the name of SRCC to carcinoma with signet ring cell differentiation in the new edition of the “WHO Classification of Breast Tumors” and no longer as an independent type [6]. Our study reported that breast cancer with signet ring cell differentiation can accompany ductal carcinoma, lobular carcinoma, and mucinous carcinoma, but the proportion of signet ring cells varies. Then, the question comes again. There is no unified conclusion about the proportion of signet ring cells that can be diagnosed as cancer with signet ring cell differentiation. Some scholars believe that to make a diagnosis of cancer with signet ring cell differentiation, signet ring-like cells must account for ≥ 10%, and the percentage of signet ring cells should be indicated in the report and that this may be related to poor prognosis. Another study compared three different cutoff values of 20%, 30%, and 40% and believed that 30% could be better related to clinical parameters [8]. The five cases in this paper have been strictly selected. Although the proportion of signet ring cells in these 5 cases ranged from 10 to 80%, the morphology of the cells fully conformed to the definition of the WHO 2012 version and was confirmed to contain intracellular mucus by AB-PAS staining.

Most primary breast cancers with signet ring cell differentiation have a breast mass as the primary symptom. Compared with other common types of invasive breast cancer, cancer with signet ring cell differentiation is more aggressive, has a higher rate of lymph node metastasis, and has a poor prognosis [9, 10]. Our cases showed that all 5 patients were admitted with breast lumps of different sizes, and two patients had obvious clinical symptoms with redness, pain, and nipple discharge. In two of these cases, vascular invasion and metastases to lymph nodes were found. Although we strictly selected all the cases, the nuclei of the signet ring cells still had various characteristics. Some cases had the same nuclear morphology, and some cases had large differences in the nuclei, with deeper staining, more intense chromatin staining, and different nuclear grades; there were also differences in the arrangement. In some cases, the adhesion between the cells was very poor, and the cells are scattered, while in some cases, the cells were arranged in sheets, clusters, or cords; similarly, the interstitium between the cells was also different, and some were rich in external mucus, and some were divided by fibrous stroma. Whether these differences are closely related to the prognosis of the tumors or due to the lack of our case data, further study of case accumulation is needed.

Molecular subtypes of breast cancer have important implications for the individualized treatment of patients. Our report shows that primary breast cancers with signet ring cell differentiation almost always express ER, PR expression is variable, and HER2 is negative in all the cases; therefore, primary breast cancers with signet ring cell differentiation are either luminal A or luminal B, and the positive index of Ki-67 also reflects this. However, because one of the cases of luminal type A had a large tumor (9.5 cm in diameter), vascular invasion, and lymph node metastasis occurred. The staging was late, and death occurred after 8 months of follow-up. This may also indicate that primary breast cancer with signet ring cell differentiation, even if it is luminal A, is still more aggressive and has a worse prognosis.

It is also crucial in distinguishing signet ring cell carcinoma from breast tissue primary from other sites. Regardless of where SRCC originates, it often metastasizes to regional lymph nodes, the peritoneal surface, ovaries, and lungs [11]. Therefore, this paper discusses the identification of primary breast cancer with signet ring cell differentiation from tumors derived from the digestive tract, lung, ovary, and other organs. First, in terms of epithelial and myoepithelial expression, CK20 is usually expressed in gastrointestinal adenocarcinoma and ovarian mucinous tumors, while SRCCs are all negative, which has identification significance. Since CK7 is expressed in most SRCCs and negatively expressed in gastrointestinal adenocarcinomas, it also has identification significance, and due to the different cell morphology of CK7 in signet ring cells, the abundant mucus in the cells may weaken its expression, but further research is needed. The myoepithelial markers CK5/6 were of little value in identifying the source. Primary breast tumors have more specific markers, such as GATA3 and GCDFP-15, and the former has a higher positive rate and is more meaningful in differentiating tumors from other sites, which is consistent with the results of previous studies [12, 13]. However, GCDFP-15 is not completely useless. Although a small number of lung adenocarcinomas may also express it, it is still meaningful to differentiate it from some tumors, such as those of the gastrointestinal tract and ovary, when it is positively expressed in breast cancer. Our results show that the specific markers of gastrointestinal tumors, including CDX2 and SATB2, were consistently negative in SRCC, while Villin or COX2 had positive expression levels in one case, so this indicator needs to be carefully considered in the differential diagnosis. In the differentiation of tumors from the ovary, PAX8 is consistently negative in SRCC, while WT-1 is partially positive, so the latter is of little significance and cannot be used alone. The related markers of lung adenocarcinoma, including TTF1 and Napsin A, were both negative in SRCC, indicating that these two indicators are of great significance in the differential diagnosis. In conclusion, in differential diagnosis, we need to pay attention to particularly useful markers, such as ER, CK20, GATA3, CDX2, SATB2, PAX8, TTF1, and Napsin A, as well as to markers whose expression is not constant, and special care should be taken when these markers are expressed abnormally.

Neuroendocrine differentiation is more likely (up to 30%) in nonspecialized invasive breast cancers or other specialized types, especially mucinous carcinomas. Therefore, this paper also observed the expression of neuroendocrine markers such as Syn, CgA, and CD56 and found that one case expressed three neuroendocrine markers. This indicates that SRCC can also be associated with neuroendocrine differentiation. In general, breast cancer with neuroendocrine features has a worse prognosis than other types of breast cancer because neuroendocrine differentiation itself is an independent poor prognostic factor [14]. Therefore, SRCC with neuroendocrine differentiation is also a problem that requires attention.

Mucins are divided into three groups according to their physiological characteristics: secreted, membrane-bound, and soluble mucins. The most typical representative membrane-bound mucin is MUC1. Ohashi et al. [8] detected the expression of various MUC proteins in breast cancer with signet ring cell differentiation and found that MUC1 expression was divided into two modes: luminal margin plus cytoplasmic positive (LC) and cytoplasmic staining with cell membrane enhanced staining (MC), where CM patterns are often associated with poor clinicopathological factors. Our study showed that MUC1 was positively expressed in all the cases, but the expression pattern was different among the cases. Some cases had weakened expression intensity, some cases had a strong perimembranous expression, and the expression level was significantly higher than that of other types of invasive cancers accompanying the surroundings. These differences are likely to be related to the clinicopathology of tumors. However, due to the small sample size included in this study, effective statistical analysis cannot be performed, and further research is needed to increase the sample size in the future. As a representative secreted mucin, MUC2 is mainly expressed in colorectal goblet cells and colon and rectal cancer cells. Walsh et al. reported that MUC2-positive breast cancer patients had significantly shorter survival than patients with MUC2-nonexpressing tumors (49 months vs. 75 months) [15]. Astashchanka Anna et al. reported that MUC2 plays an important role in mediating breast cancer cell proliferation, apoptosis, and metastasis. MUC2 may be important to guide treatment and predict outcomes in breast cancer patients [16]. Our experimental results showed that MUC2 was strongly expressed in all the cases and was significantly higher than that in the peripheral invasive ductal carcinoma, lobular carcinoma, or mucinous carcinoma. Whether this is associated with a worse prognosis in breast cancers with signet ring cell differentiation remains to be investigated.

In addition, the results of AB (pH 2.5)-PAS staining in all the cases showed that breast signet ring cell carcinoma had greater heterogeneity, which not only manifested in the mixed existence of multiple histological morphologies in the same tumor tissue but also showed differences in the properties and amount of intracellular mucus in similar tumor tissues in the different cases or different mucus lakes in the same tumor tissue. We can see neutral mucus, acidic mucus, mixed mucus, and transitional morphologies in all the cases. Different signet ring cell carcinomas are either predominantly neutral mucus (red cells), acidic mucus (blue cells), or both (purple cells), but neutral mucus is generally the most common. The main signet ring cells are more abundant, and the extracellular mucus is more acidic. Yameshina [17] confirmed that cancer cells have diverse shapes and different mucus reactions, indicating that cancer cells are in different stages. We believe that signet ring cell carcinoma has multiple cell subsets and retains the potential for multidirectional differentiation. The difference in mucus expression and traits of cancer cells may be due to the differentiation and development of stem cells at different stages or different cloned tumor cells. Thus, during tumor evolution, cancer cells express different mucinous traits. In addition, it is speculated that the influence of precancer environmental factors or stress also plays an important role in the heterogeneity of cellular mucus, which is also required for tumor cell phenotypes to change to suit their survival [18].

In conclusion, although breast cancer with signet ring cell differentiation is no longer regarded as an independent type, these tumors still have their own characteristics, and their histological morphology is still different even if they are strictly defined. The molecular type is mainly luminal A or type B, and their expression of some immune markers is inconsistent with that of the surrounding ductal, lobular, and mucinous carcinomas. We need to be especially cautious when aberrant expression of nonconstant markers is used in differential diagnosis. The expression pattern of mucin markers may be related to the clinicopathological characteristics of tumors, such as tumor prognosis, and the characteristics of mucin in these tumor cells may also be closely related to the occurrence and evolution of tumors.

## Data Availability

Please contact the corresponding author with a request for data.

## Abbreviations

SRCC: Signet ring cell carcinoma
AB-PAS: Alcian Blue (pH 2.5)-Periodic Acid-Schiff
IHC: Immunohistochemistry
ER: Estrogen receptor
PR: Progesterone receptor
HER2: Human epidermal growth factor receptor 2
GCDFP-15: Gross cystic disease fluid protein-15
SATB2: Special AT-rich sequence-binding protein 2
GATA-3: GATA binding protein 3
CDK4: Cyclin-dependent kinase 4
COX-2: Cyclooxygenase 2
WT-1: Wilm’s tumor gene 1
PAX-8: Pair box gene
TTF-1: Thyroid transcription factor-1
Syn: Synapsin
CgA: Chromogranin A
EGFR: Epidermal growth factor receptor
